# Cemented versus Cementless Stem Fixation in Revision Total Knee Arthroplasty: A Systematic Review and Meta-Analysis

**DOI:** 10.3390/antibiotics12111633

**Published:** 2023-11-17

**Authors:** Ali Darwich, Andrea Jovanovic, Franz-Joseph Dally, Asseel Abd El Hai, Tobias Baumgärtner, Elio Assaf, Sascha Gravius, Svetlana Hetjens, Mohamad Bdeir

**Affiliations:** 1 Department of Orthopaedic and Trauma Surgery, University Medical Centre Mannheim, Medical Faculty Mannheim, University of Heidelberg, 68167 Mannheim, Germany; andrea.jovanovic@medma.uni-heidelberg.de (A.J.); franz.dally@umm.de (F.-J.D.); asseel.abdelhai@umm.de (A.A.E.H.); tobias.baumgaertner@umm.de (T.B.); sascha.gravius@umm.de (S.G.); mohamad.bdeir@umm.de (M.B.); 2 Department of Orthopaedic and Trauma Surgery, University Hospital Bonn, 53127 Bonn, Germany; elio.assaf@ukbonn.de; 3 Institute of Medical Statistics and Biomathematics, University Medical Centre Mannheim, Medical Faculty Mannheim, University of Heidelberg, 68167 Mannheim, Germany; svetlana.hetjens@medma.uni-heidelberg.de

**Keywords:** revision total knee arthroplasty, stem, fixation, cemented, cementless, failure, revision, clinical outcome

## Abstract

The number of revision knee arthroplasties (rTKA) is growing significantly as is the use of intramedullary stems for optimized stability. The choice of the most appropriate stem fixation method is still controversial. The purpose of this meta-analysis is to compare cemented versus cementless stem fixation in rTKA. Publications with patients undergoing rTKA with a follow-up > 24 months were systemically reviewed. Extracted parameters included total revision and failure rates for any reason, incidence of aseptic loosening, periprosthetic infection, and radiolucent lines, as well as the clinical outcome. A statistical regression analysis was then performed on all extracted clinical and radiological outcome data. A total of 35 publications met the inclusion criteria and were included and analyzed. Overall, 14/35 publications compared cementless versus cemented stem fixation, whereas 21/35 publications investigated only one stem fixation method. There were no significant differences in revision (*p* = 0.2613) or failure rates (*p* = 0.3559) and no differences in the incidence of aseptic loosening (*p* = 0.3999) or periprosthetic infection (*p* = 0.1010). The incidence of radiolucent lines was significantly higher in patients with cemented stems (26.2% versus 18.6%, *p* < 0.0001). However, no differences in clinical outcomes were observed. No superiority of a specific stem fixation method in rTKA was found. Rates of revision or failure for any reason as well as incidence of aseptic loosening and periprosthetic infection in cemented versus cementless stem fixation showed no significant difference. A higher incidence of radiolucent lines was observed in cemented stem fixation; however, no effect was observed on the clinical outcome.

## 1. Introduction

Due to the demographic changes in recent years and the continuously ageing society as well as the growing demand of an active lifestyle, the number of performed total joint arthroplasties (TJA) is increasing significantly [1,2]. At the same time, the age of the patients undergoing those surgeries is decreasing, meaning that considerably higher numbers of revisions in total joint arthroplasty are to be expected [3].

The continuously optimized surgical techniques on one side and unremitting development of material and designs of modern implants on the other has led to a radical improvement of results in primary total knee arthroplasty (TKA) with good outcomes in 90–95% of cases at 10–15 years of follow-up and implant survival rates greater than 90% [4]. Though the same efforts are being made in revision total knee arthroplasty (rTKA), outcomes remain worse in comparison to primary TKA with significantly higher failure rates [5]. Geary et al. [6] reported failure rates of 22.8% at a minimum follow-up of 2 years in patients with rTKA. In their study, septic failure was reported in 38.5% of cases, and aseptic failure in 61.5%. In cases of aseptic failure, aseptic loosening was reported to be the most common cause (20.9%), followed by instability (14.2%), stiffness (4.5%), periprosthetic fracture (3.5%), and wear/osteolysis (2.9%) [6].

Some of the main challenges in rTKA are the management of bone loss, osteopenic bone structure, and deformity [3]. Intramedullary stems are in this context a valuable tool to allow stress transfer and enhance stability. Both cemented and cementless intramedullary stems are available: advocates of the cemented option report a better metaphyseal coverage with an antibiotic-laden capacity [7], whereas the supporters of the cementless alternative report better component alignment and less stress shielding [8,9]. Based on the limited data available, the superiority of one of the two stem fixation methods remains controversial [3]. 

Thus, the purpose of this meta-analysis was to compare cemented versus cementless stem fixation in rTKA regarding implant survivorship, failure rates and its causes, as well as clinical outcome of each of these fixation methods.

## 2. Materials and Methods

This study was conducted according to the PRISMA (Preferred Reporting Items for Systematic Reviews and Meta-Analyses) checklists and guidelines [10]. The systematic review was not registered beforehand and a protocol in advance was not prepared.

### 2.1. Search Strategy

A systematic literature search strategy was applied to the following databases: PubMed, Ovid Medline, the Cochrane Library, Web of Science, and PsycInfo (via EBSCO). The PICO Model was used while performing the search [11]. 

The search was performed using the MESH terms “Reoperation, Revision” or a combination of the following keywords:

“Total Knee Replacement*” OR “Total Knee Arthroplasty*”
AND
“Reoperation” OR “Revision” OR “Reimplantation”
AND
“stem*” OR “cement*” OR “hybrid”.

Publications in foreign languages relevant to the meta-analysis (excluding English) were translated and then included in the statistics if the inclusion criteria were met.

The search was performed by a qualified medical librarian and revised/completed on 1 August 2023.

### 2.2. Study Selection and Eligibility Criteria

There were no language limitations in the selection of the articles. Exclusion criteria included follow-up of less than 24 months, experimental studies on animals or cadavers, studies including patients with malignant bone tumors without providing a separate analysis of the remaining patients, case reports, and surgical techniques, and/or overviews of treatment options. 

The identified articles were screened by two of the authors independently (A.J., A.A.E.H.) through reviewing the title and abstract of each study. Inclusion of the relevant articles was completed after reading the full text and identifying the required parameters. Furthermore, reference lists of the selected studies were inspected for additional relevant articles. Systematic reviews and meta-analyses were then screened to check whether they contained potential studies that had not been included in the literature search.

### 2.3. Data Collection Process

In the analysis, emphasis was placed on the number of knees or stems rather than on the cohort size, as this has a higher relevance in the investigation of stem fixation. There were no automation tools used in the data collection process. Data collection was performed by two of the authors (A.J., A.A.E.H.) and validated by another four authors (F.-J.D., A.A.E.H., T.B., E.A).

In addition to the type of stem fixation (cementless versus cemented), the collected data included the total revision rates and total failure rates for any reason as well as the incidence of aseptic loosening, periprosthetic infections, instability regardless of the level of constraint, periprosthetic fractures, and development of radiolucent lines. Clinical outcomes were also recorded through the clinical Knee Society Score (cKSS), functional Knee Society Score (fKSS), total Knee Society Score (tKSS), Western Ontario and McMaster Universities Osteoarthritis Index (WOMAC), and range of motion (ROM). Furthermore, the sample size, the average follow-up, the reason for revision or re-revision, the gender distribution, and the age of the patients were recorded.

The data were then categorized and ordered depending on the stem fixation type. Further details about the implant, such as design (modular, monoblock, etc.), size, manufacturer, insert type, level of constraint, or canal-fill ratio, were not taken into account.

### 2.4. Study Risk of Bias Assessment

The included studies were evaluated for methodological flaws using the Cochrane Collaboration’s risk of bias assessment tool (Review Manager version 5.3). Seven domains of risk of bias were assessed for each study, including random sequence generation, allocation concealment, blinding of participants and personnel, blinding of outcome assessment, incomplete outcome data, selective reporting, and other bias. Bias assessment was performed by two of the authors independently (A.J., A.A.E.H.). Validation and further formatting in the above-mentioned assessment software was performed by a third author (S.H.).

### 2.5. Effect Measures

The results of the different studies and their main outcome parameters including revision, failure, aseptic loosening, periprosthetic infection, radiolucent lines, and instability, with 95% confidence interval (CI), and the pooled proportion with 95% CI were shown in a forest plot. The Chi^2^ test was used to determine the relative risk in order to subsequently derive the significance of the differences between the different fixation options. The Fisher test was used to analyze the clinical scores (KSS, WOMAC, range of motion).

### 2.6. Synthesis Methods and Statistical Analysis

For the statistical analysis, the programs MedCalc (MedCalc^®^ Statistical Software version 20.111, MedCalc Software Ltd., Ostend, Belgium) and SAS (SAS software, release 9.4, SAS Institute Inc., Cary, NC, USA) were used. The random effect model was calculated in the meta-analysis. MedCalc uses a Freeman–Tukey transformation (arcsine square root transformation) [12] to calculate the weighted summary proportion under the random effects model [13]. A Cochran’s Q and I^2^ index were used to assess heterogeneity between studies included in the analysis. Q is the weighted sum of squares on a standardized scale. A low *p*-value of the Cochran’s Q statistic indicates presence of heterogeneity. I^2^ interpretation according to Higgins [14]: I^2^ = 0%: there is no observed heterogeneity; I^2^ > 0% and ≤ 25%: there is insignificant heterogeneity; I^2^ > 25% and ≤ 50%: there is low heterogeneity; I^2^ > 50% and ≤ 75%: there is moderate heterogeneity; and I^2^ > 75%: there is high heterogeneity. The weighted Chi^2^ test was used to determine the relative risk in order to subsequently derive the significance of the differences between the different fixation options (SAS Procedure PROC FREQ with option WEIGHT for number of knees). The weighted *t*-test was used to analyze the clinical scores. A *p*-value of < 0.05 was considered a statistically significant option (SAS Procedure PROC TTEST with option WEIGHT for number of knees).

## 3. Results

### 3.1. Search Results

After applying the search strategy described under “Materials and Methods”, 3051 articles were identified. Overall, 1042 records were duplicates and were removed before screening, 2009 articles were screened, and ultimately 40 articles were assessed for final eligibility. In total, 6 articles recorded the investigated outcome measures insufficiently and had to be excluded, leaving 34 studies for inclusion in the meta-analysis. 

**Figure 1:**
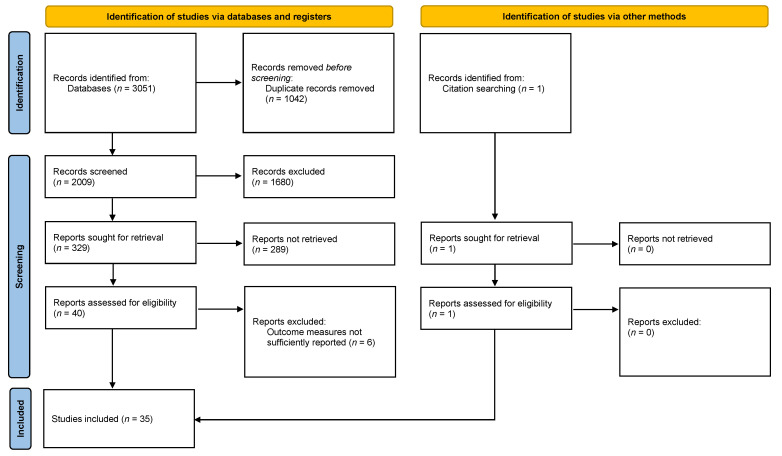
Study selection flow diagram [15].

After screening systematic reviews and meta-analyses, one more article was found and included. In the final meta-analysis, 35 publications were included (Figure 1) and further analyzed. 

### 3.2. Study Characteristics

Of the included 35 publications, 14 compared cemented to cementless stem fixation in rTKA. The remaining 21 studies investigated only one of the two mentioned stem fixation methods: 4 studies involved only patients with cemented stem fixation and 17 studies involved only patients with cementless stem fixation. A total of 3203 knees or stems were examined, of which 831 belonged to the group with cemented stem fixation and 2372 to the group with cementless stem fixation. The included patients were available for a mean follow-up of 57.5 ± 26.4 months (range 18–122 months) (Table 1).

The mean age of all patients included in the meta-analysis was 66 ± 10.2 years (range 20–82.6 years); 72% of the included patients were females and 28% were males. Age and female/male distribution of the patients as well as follow-up periods did not significantly differ between the studied groups.

### 3.3. Risk of Bias in the Included Studies

The bias analysis of the included studies showed a high selection bias (random sequence generation) and high performance bias each in 12 of the 35 included studies. A high detection bias was only found in three studies. Attrition bias was found in 7 of the 35 included studies. 

**Figure 2:**
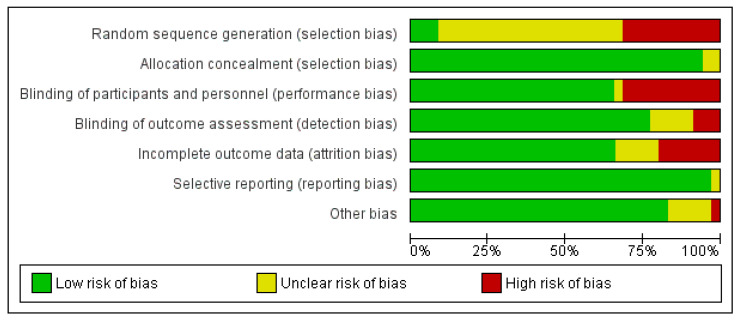
Methodological quality of the studies included in the meta-analysis.

The total results of the bias evaluation of all studies were presented in the risk of bias graph (Figure 2).

### 3.4. Results of Individual Studies

No superiority of a specific stem fixation method in rTKA was found in this meta-analysis. 

**Figure 3:**
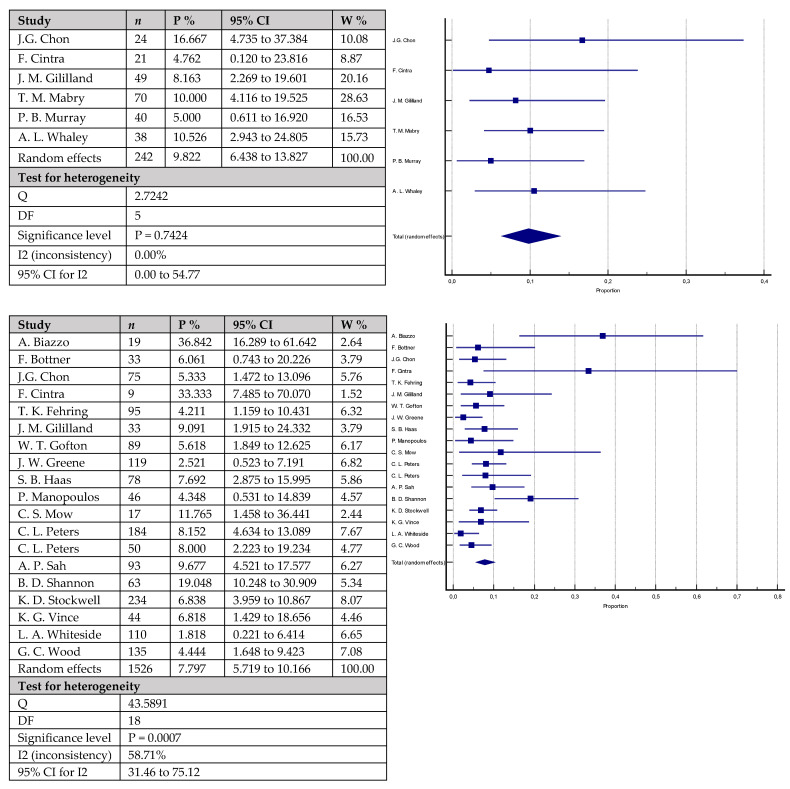
Forest plots showing revision rates for any reason. Upper chart represents data from studies involving patients with cemented stem fixation and lower chart represents data from studies with cementless stem fixation (n—sample size, P %—Proportion, CI—Confidence interval, W %—Weight (Random)) [7,18,19,20,21,23,25,26,28,29,36,37,39,40,41,42,43,44,45,46,47,49].

**Figure 4:**
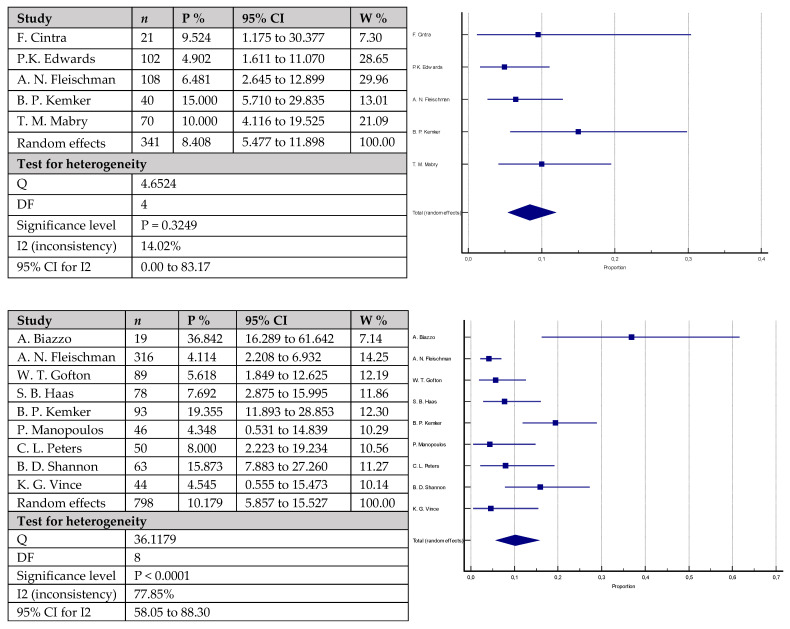
Forest plots showing failure rates for any reason. Upper chart represents data from studies involving patients with cemented stem fixation and lower chart represents data from studies with cementless stem fixation (*n*—sample size, P %—Proportion, CI—Confidence interval, W %—Weight (Random)) [18,21,22,24,26,29,32,36,37,41,43,45].

Regarding total revision rates for any reason (Figure 3), the group involving patients with a cemented stem fixation showed rates of 9.8% (95% confidence interval, CI = 6.428–13.827) in 242 knees versus 7.8% (95% CI = 5.719–10.166) in 1526 knees in the group with patients with cementless stems. The difference was not statistically significant (*p* = 0.2613). The observed difference in total failure rates for any reason (Figure 4) between the two studied groups (8.4% (95% CI = 5.477–11.898) in 341 knees with cemented stems versus 10.2% (95% CI = 5.857–15.527) in 798 knees with cementless stems) was also not statistically significant (*p* = 0.3559).

**Figure 5:**
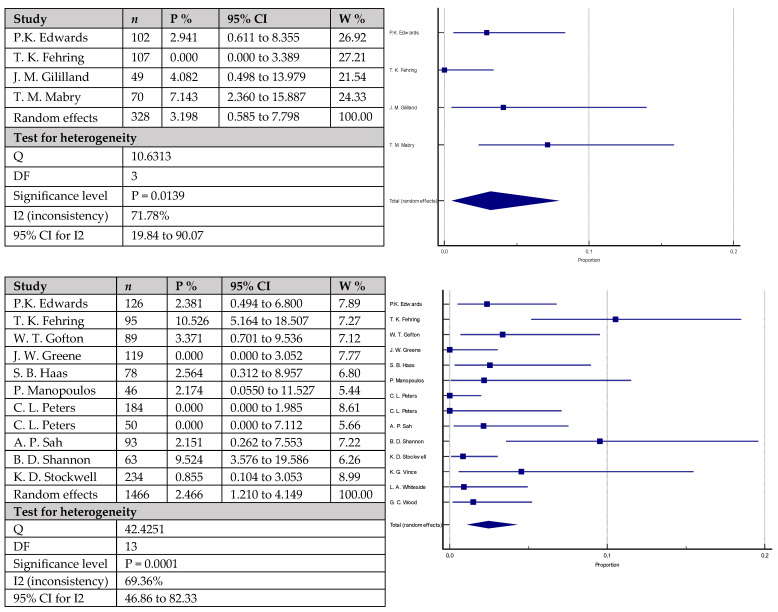
Forest plots showing rates of aseptic loosening. The upper chart represents data from studies involving patients with cemented stem fixation and the lower chart represents data from studies with cementless stem (*n*—sample size, P %—Proportion, CI—Confidence interval, W %—Weight (Random)) [22,23,25,26,28,29,36,37,40,41,42,43,44].

**Figure 6:**
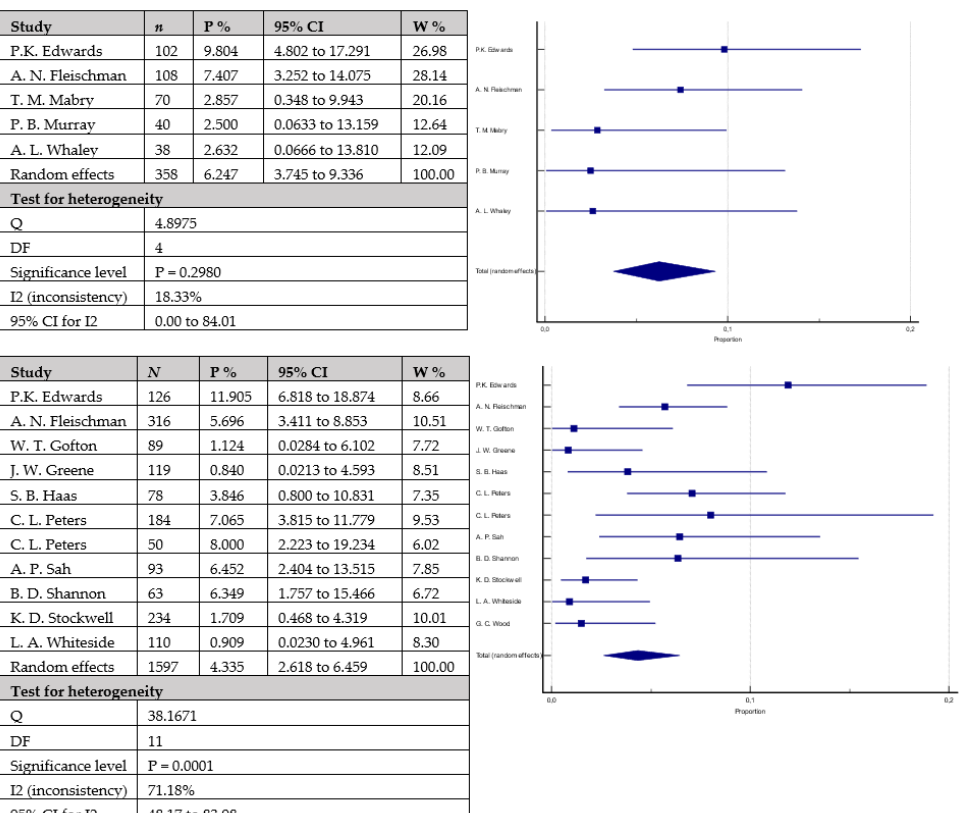
Forest plots showing rates of periprosthetic infections. The upper chart represents data from studies involving patients with cemented stem fixation and the lower chart represents data from studies with cementless stem fixation (*n*—sample size, P %—Proportion, CI—Confidence interval, W %—Weight (Random)) [7,22,24,26,28,29,36,40,41,42,43,44,46,47].

**Figure 7:**
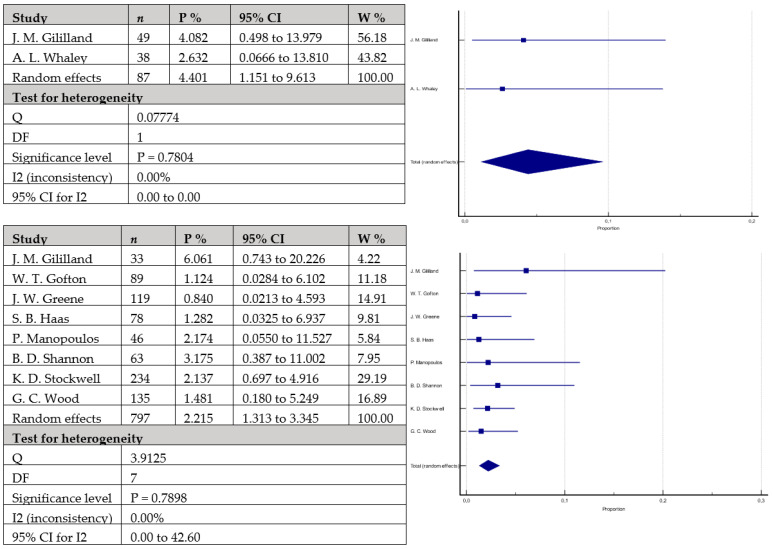
Forest plots showing rates of instability. The upper chart represents data from studies involving patients with cemented stem fixation and the lower chart represents data from studies with cementless stem fixation fixation (*n*—sample size, P %—Proportion, CI—Confidence interval, W %—Weight (Random)) [25,26,28,29,37,43,44,46,49].

The rates of aseptic loosening (Figure 5) were 3.2% (95% CI = 0.585–7.798) in 328 knees with cemented stems versus 2.5% (95% CI = 1.210–4.149) in 1466 knees with cementless stems. The difference did not reach statistical significance (*p* = 0.3999). Similarly, the rates of periprosthetic infections (Figure 6) were mildly higher in the group with cemented stems with 6.3% (95% CI = 3.745–9.336) of the 358 included knees versus 4.3% (95% CI = 2.618–6.459) of the 1597 included knees with cementless stems, without reaching statistical significance (*p* = 0.1010). Equally to the above-mentioned parameters, joint instability rates (Figure 7) did not statistically vary between the studied groups: 4.4% (95% CI = 1.151–9.613) in the group with cemented stems (*n* = 87) versus 2.2% (95% CI = 1.313–3.345) in the cementless group (*n* = 797) (*p* = 0.2621). 

**Figure 8:**
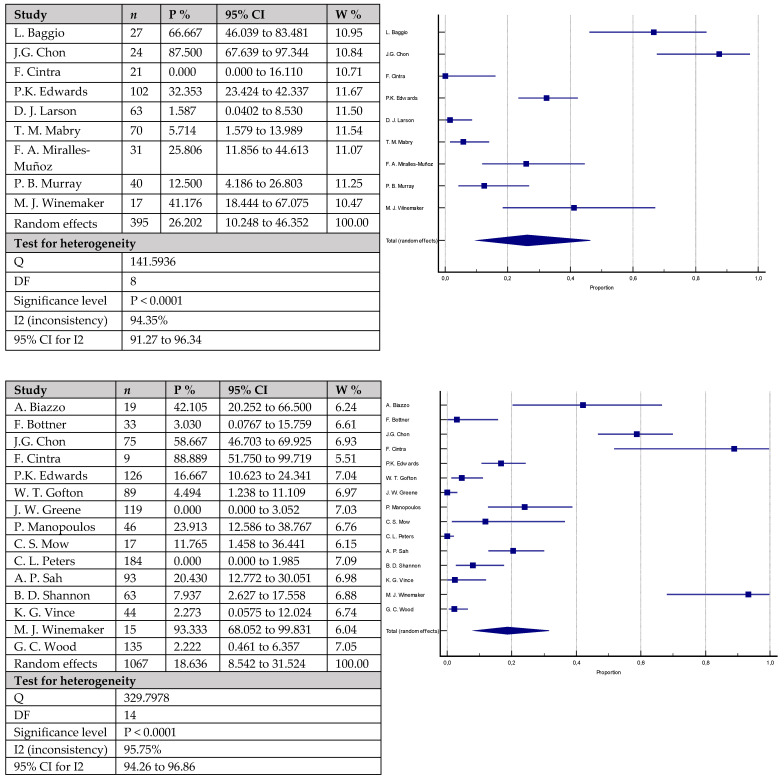
Forest plots showing incidence of radiolucent lines. The upper chart represents data from studies involving patients with cemented stem fixation and the lower chart represents data from studies with cementless stem fixation fixation (*n*—sample size, P %—Proportion, CI—Confidence interval, W %—Weight (Random)) [7,16,18,19,20,21,22,26,28,35,36,37,38,39,40,42,43,45,48,49].

The only statistically significant difference between the studied groups was the incidence of radiolucent lines (Figure 8), where an incidence of 26.2% (95% CI = 10.248–46.352) was observed in the group including 395 knees with cemented stems versus 18.6% (95% CI = 8.542–31.524) in the second group with 1067 knees with cementless stems (*p* < 0.0001).

Despite the higher incidence of radiolucent lines in patients with cemented stems, there were no statistically significant differences between the two groups concerning clinical scores. The Knee Society Score (KSS) did not significantly vary with 138.15 ± 12.89 points in the first group with cemented stems versus 149.47 ± 16.22 points in the second group (*p* = 0.3780). The Western Ontario and McMaster Universities Osteoarthritis Index (WOMAC) was also not significantly different between the two groups (75.65 ± 8.03 points in the cemented group versus 82.36 ± 3.01 points in the cementless group, *p* = 0.3235). Similarly, there were no significant differences regarding range of motion with mean 96.09 ± 8.32 degrees in the cemented group and 101.75 ± 5.32 degrees in the cementless group (*p* = 0.1036). Further details are found in the summary of the compared parameters between the different stem fixation groups in Table 2.

Further parameters such as periprosthetic fractures were not presented as the available data were insufficient for a comparison and statistical significance.

## 4. Discussion

Based on the results of the presenting meta-analysis, no evidence of superiority of neither the cemented nor the cementless stem fixation technique in rTKA could be found. The results of this study identified no significant differences in revision rates, failure rates, rates of aseptic loosening, periprosthetic infections, or instability between the two stem fixation methods. A significantly higher incidence of radiolucent lines in knees with cemented stems was observed. However, there were no significant differences in clinical outcome, which further supports comparable surgical outcomes between cemented and cementless stems in rTKA.

That the majority of orthopedic surgeons would prefer the cementless stem fixation [3] is supported by the fact that 2372 (74%) of the total number of included knees (*n* = 3203) in this meta-analysis belonged to the group with cementless stem fixation and 831 (26%) to the group with cemented stems. However, this tendency could not be supported by the data of the presenting meta-analysis. 

Intramedullary stems in the setting of rTKA have several advantages in the restoration of joint stability and providing a correct component alignment, which eventually improves the durability of the revised implant [9,50]. Their use in rTKA, especially the modular stem design, is favored despite the risk of junctional failure. In fact, higher failure rates have been reported in rTKA cases without the use of intramedullary stems, where failure rates increased from 8% with stem usage to as high as 66% without stems at 5-year follow-up [51,52]. However, the choice of the most appropriate fixation technique remains a challenging decision [32]. 

Cemented stems have been reported to provide sufficient primary stability with stems as short as 30 mm [16,32,46,53,54]. They offer flexibility and can compensate bone defects and bone loss as well as bone canal deformities and irregularities [55]. They may also have advantages in reducing intraoperative blood loss [32] but have the downside of higher incidence of stress shielding and increased bone loss in cases where an explantation of the prosthesis is needed [8,56]. 

When using bone cement in the cemented stem fixation, the addition of antibiotics can offer the advantage of a high local antibiotic activity compared to systemic administration [57]. However, one drawback is the possible bone cement implantation syndrome (BCIS), which is considered to be a potentially fatal complication [58]. Rassir et al. [59] reported a 28% incidence of BCIS in primary knee arthroplasties and 23% in revision arthroplasties. Manifestations may vary from mild hypoxia to severe cardiovascular collapse necessitating CPR [60]. The underlying exact pathomechanism behind BCIS is not well understood; however, Moldovan et al. [61] suggested multiple factors that play a role in the pathophysiology in the sense of a multi-modal theory, taking into consideration the patient’s parameters as well. Some of the suggested measures to prevent BCIS included using a low viscosity cement which reduces intramedullary pressure [62], adequate intramedullary lavage prior to cementing to remove debris and achieving hemostasis [63], venting hole drilling in the canal distally to relieve pressure when inserting the stem [64], as well as the use of modern cementing techniques such as vacuum-mixing of cement and retrograde insertion using cement guns [65].

An alternative is obviously the use of uncemented implants. Cementless stems have been reported to provide better functional outcomes [19,28,66] and obviously less bone loss in case a re-revision with prosthesis explantation is needed. However, in order to achieve a correct alignment, as the components’ position may be predetermined by the position of the press-fit stem, the use of offset adapters may be necessary [32]. Cementless stems have also been reported to cause stem pain [67,68,69] and to be associated with higher rates of periprosthetic fractures [70].

Several studies examined micromotion and implant stability in the setting of rTKA using cemented or cementless stems and found no significant difference. A recent study from Mills et al. [71] investigated long-term micromotion and the corresponding 10-year stability of rTKA with cemented and cementless stems using radiostereometric analysis. The analysis showed a median total femoral translation and rotation of 0.39 mm and 0.59° for the cemented group and 0.70 mm and 0.78° for the cementless group. Regarding tibial components, the measurements were 0.38 mm and 0.98° for the cemented group and 0.42 mm and 0.72° for the cementless group. None of the differences in measurements between the two groups reached statistical significance. A similar study from Heesterbeek et al. [30] investigating micromotion in rTKA with mild to moderate bone loss also showed equal stability between the two fixation techniques.

The presenting meta-analysis shows failure rates of 10% for the cementless stem fixation and 8% for the cemented stem fixation. These results go in line with most reported results in the literature where a 9–11% failure rate was documented for the cemented fixation group and 6–10% for the cementless group [3,20,22,23,72]. 

Other studies report relatively higher failure rates [22,24,32,73]. Kemker et al. [32] reported failure rates of 17.5% and 19.4% for the cemented and cementless groups, respectively, after a mean follow-up of 25.8 months. The authors state that the study took place at a tertiary referral center with a complex patient population, which may have had an influence on the failure rates [32]. The follow-up period also has an effect on the reported rates. Fleischman et al. [24] reported 5-year mechanical failure rates of 3.5% and 5% for the cemented and cementless groups, respectively. At 10 years follow-up, mechanical failure rates increased to 17.1% and 22.8% for the cemented and cementless groups, respectively. Similarly, Leta et al. [73] reported failure rates of 15% at 5 years, 22% at 10 years, and 29% at 15 years after aseptic rTKA based on data from the Norwegian Arthroplasty Register.

A significant discrepancy in the rate of radiolucent lines between patients with cemented vs. cementless stem fixation was found in the presenting meta-analysis. Radiolucent lines were found in 26.2% of patients with cemented stem fixation versus 18.6% of patients with cementless stems (*p* < 0.0001). Despite this significant discrepancy, there were no statistically significant differences between the two groups concerning clinical scores including KSS, WOMAC and range of motion. This goes in line with literature data confirming the absence of correlation between appearance of radiolucencies or radiolucent lines and clinical scores or implant survival [28,38,40,43].

Several previous studies investigated the appearance of radiolucencies around the used stems in the setting of revision knee arthroplasty and reported a wide range of discrepancy with rates from 19% to up to 74% [17,23,29,43,74]. Modern cementing techniques and optimized femoral/tibial canal fill as well as modern implant designs, especially long fluted stems, are thought to play a role in the decreasing rates of these radiolucencies in cemented stems [28,40]. On the other hand, stem micromotion and aggressive canal preparation are theories that may explain the appearance of radiolucencies in cementless stems [26,42,75].

This meta-analysis has several limitations. 

One of the main limitations of the available data was the heterogeneity of the analyzed parameters. Some studies recorded only the rates of revision or failure, some also recorded the cause of failure, and others investigated the clinical outcome as well. In addition, the follow-up periods were heterogeneous and ranged from 18 months to up to 127 months. 

While the total number of patients included in this meta-analysis is adequate, another limitation is the relatively small sample size of some of the included studies. Nonetheless, the mean values and standard deviations given in each study were weighed according to the number of patients. 

A third limitation is the fact that further details about the included implants, such as design incl. monoblock or modular designs, size, level of constraint, manufacturer, insert-type, or canal-fill ratio, were not taken into account. Only few studies recorded these details, which made a subgroup analysis impossible. However, this may have acted as a confounder. 

The design of the implant used and especially the material used is of great significance. Besides junctional failure of modular implants [76], another possible complication of cementless implants is hardware failure, notably the tibial component. Scully et al. [77] and Fokter et al. [78] investigated one catastrophic complication involving complete failure of uncemented porous tantalum tibia components in primary knee arthroplasty. These components were thought to reduce backside wear and polyethylene debris. However, short- to medium-term follow-up showed failure of the tibial baseplate with insufficient biologic ingrowth and loss of structural bone support, leading to subsequent fracture and dislocation of the component [78]. In both cases reported, the fracture occurred at the junction between supported and non-supported areas of the baseplate. In rTKA, metaphyseal bone loss in the tibia is a frequent problem [79]. One of the available options for defect management include tantalum trabecular metal tibial cones [80]. Meneghini et al. [81] reported good short-term outcome for porous tantalum metaphyseal cones in patients with severe tibial bone loss, especially with no evidence of migration or loosening, and Kamath et al. [82] reported revision rates of less than 5% at six years for the same cones. Nonetheless, special care should be given when using such defect management alternatives, since long-term results are still missing.

## 5. Conclusions

No superiority of a specific stem fixation method in rTKA was found. Rates of revision or failure for any reason as well as incidence of aseptic loosening and periprosthetic infection in cemented versus cementless stem fixation showed no significant difference. A higher incidence of radiolucent lines was observed in cemented stem fixation; however, with no effect on the clinical outcome. 

## Figures and Tables

**Table 1:** Details of the studies included in the meta-analysis.

Study	Year	Fixation	Antibiotic-Impregnated Cement?		No of Knees	Mean Age (Years)	Male %	Female %	Follow-up (Months)
L. Baggio [16]	2016	c	Yes	Gentamicin	27	82.6	33.3	66.7	43
K.C. Bertin [17]	1985	u	-	-	53	-	32.1	67.9	18
A. Biazzo [18]	2022	u	-	-	19	69.9	21.1	78.9	33.6
F. Bottner [19]	2006	u	-	-	33	68	36.4	63.6	38
J.G. Chon [20]	2004	c	n/a	n/a	24	65	-	-	44
		u	-	-	75	65	-	-	44
F. Cintra [21]	2011	c	Yes	Gentamicin (1 g per dose)	21	62.8	46.2	53.8	62
		u	-	-	9				42
P.K. Edwards [22]	2014	c	Yes	Not recorded	102	65	38.5	61.5	45
		u	-	-	126	65	49.2	50.8	52
T. K. Fehring [23]	2003	c	Yes	Not recorded	107	67.5	38.5	61.5	53
		u	-	-	95				61
A. N. Fleischman [24]	2017	c	Yes	Not recorded	108	65.8	30.6	69.4	64.3
		u	-	-	316	63.9	42.7	57.3	59.6
J. M. Gililland [25]	2014	c	Yes	Not recorded	49	65	49	51	76
		u	-	-	33	64	33.3	66.7	121
W. T. Gofton [26]	2002	u	-	-	89	69.1	36	64	70.8
J. Gómez-Vallejo [27]	2018	c	No	-	29	79.7	-	-	84
		u	-	-	38	78.4	-	-	
J. W. Greene [28]	2013	u	-	-	119	67	53.8	46.2	62
S. B. Haas [29]	1995	u	-	-	78	61	35.9	64.1	42
P. J. Heesterbeek [30]	2016	c	Yes	Gentamicin Gentamicin/Clindamycin Gentamicin/Vancomycin	15	67	37.5	62.5	24
		u	-	-	15	64.5	18.7	81.3	
M. M. Iamaguchi [31]	2013	u	-	-	35	68.5	40	60	26.4
B. P. Kemker [32]	2022	c	n/a	n/a	40	63.8	32.5	67.5	24.6
		u	-	-	93	63.8	36.6	63.4	24.6
N. M. Kosse [33]	2017	c	Yes	Gentamicin Gentamicin/Clindamycin Gentamicin/Vancomycin	12	73	33.3	66.7	78
		u	-	-	11	67	20	80	
P. F. Lachiewicz [34]	2020	c	Yes	Gentamicin/Tobramycin	34	68	38.2	61.8	72
		u	-	-	50	68	38	62	
D. J. Larson [35]	2021	c	n/a	n/a	63	65.9	47.6	52.4	60
		u	-	-	47	66	57.4	42.6	
T. M. Mabry [36]	2007	c	Yes	Not recorded	70	73	55.7	44.3	122.4
P. Manopoulos [37]	2012	u	-	-	46	69	37	63	102
F. A. Miralles-Muñoz [38]	2022	c	Yes	Not recorded	31	67.8	38.7	61.3	75.6
		u	-	-	42	65.3	40.5	59.5	75.6
C. S. Mow [39]	1994	u	-	-	17	65	58.8	41.2	72
P. B. Murray [7]	1994	c	n/a	n/a	40	67.2	47.5	52.5	58.2
C. L. Peters [40]	2009	u	-	-	184	63	32.6	67.4	48
C. L. Peters [41]	2005	u	-	-	50	68	32	68	36
A. P. Sah [42]	2011	u	-	-	93	68.8	33.3	66.7	65
B. D. Shannon [43]	2003	u	-	-	63	66	52.4	47.6	69
K. D. Stockwell [44]	2019	u	-	-	234	68	43.2	56.8	58.8
K. G. Vince [45]	1995	u	-	-	44	-	-	-	24–72
A. L. Whaley [46]	2003	c	n/a	n/a	38	67	42.1	57.9	121.2
L. A. Whiteside [47]	2006	u	-	-	110	73	40	60	60–127
M. J. Winemaker [48]	1998	c	n/a	n/a	17	71.9	33.3	66.7	28
		u	-	-	15	69.8			28
G. C. Wood [49]	2009	u	-	-	135	71	41.5	58.5	60

c cemented fixation, u cementless fixation, n/a not available.

**Table 2:** Summary of the compared parameters between the different stem fixation groups.

Parameter/Rate	Cementless Fixation		Cemented Fixation		*p*-Value
	Value	95% CI	Value	95% CI	
Revision	7.797%	5.719–10.166	9.822%	6.428–13.827	0.2613
Failure	10.179%	5.857–15.527	8.408%	5.477–11.898	0.3559
Asepticloosening	2.466%	1.210–4.149	3.198%	0.585–7.798	0.3999
Periprosthetic infection	4.335%	2.618–6.459	6.247%	3.745–9.336	0.1010
Radiolucent lines	18.636%	8.542–31.524	26.202%	10.248–46.352	<0.0001 *
Instability	2.215%	1.313–3.345	4.401%	1.151–9.613	0.2621
KSS total	149.47 ± 16.22 points		138.15 ± 12.89 points		0.3780
KSS functional	58.81 ± 12.40 points		65.51 ± 12.03 points		0.2678
KSS clinical	81.81 ± 5.06 points		76.20 ± 12.37 points		0.0841
WOMAC	82.36 ± 3.01 points		75.65 ± 8.03 points		0.3235
Range of motion	101.75 ± 5.32 degrees		96.09 ± 8.32 degrees		0.1036

CI Confidence interval, KSS Knee society score, WOMAC Western Ontario and McMaster Universities Osteoarthritis Index, * statistically significant.

## Data Availability

The data presented in this study are available on request from the corresponding author.
